# Effects of Burdock Addition and Different Starters on the Quality and Flavor Improvement of Duck Sausages

**DOI:** 10.3390/biology14080996

**Published:** 2025-08-04

**Authors:** Li Cui, Xuan Zhao, Xingye Song, Wenjing Zhou, Tao Wang, Wuyang Huang, Yuxing Guo

**Affiliations:** 1 Institute of Agro-Product Processing, Jiangsu Academy of Agricultural Sciences, Nanjing 210014, China; clisu1@163.com; 2 School of Food Science and Pharmaceutical Engineering, Nanjing Normal University, Nanjing 210023, China; zx@highbery.cn (X.Z.); 19291722396@163.com (X.S.); 13789404199@163.com (T.W.); 3 College of Life Science and Food Engineering, Huaiyin Institute of Technology, Huaian 223003, China; zhouwenjing0911@163.com

**Keywords:** duck sausage, burdock, lactic acid bacteria, off-odor, antioxidant capacity

## Abstract

Burdock (*Arctium lappa* L.) is a medicinal and edible homologous plant whose roots contain many bioactive substances such as polysaccharides and phenolics. This study explored the integration of burdock powder and lactic acid bacteria fermentation to enhance the nutritional quality, sensory attributes, and flavor profiles of duck sausages. Three bacterial strains, *Lacticaseibacillus casei*, *L. helveticus*, and *L. plantarum*, were selected based on sensory analysis, and their effects on sausage properties were evaluated through combined fermentation trials. The results demonstrated that duck sausages fermented with *L. plantarum* and *L. helveticus* and supplemented with 3% burdock powder (PHB group) exhibited > 1.5-fold higher antioxidant activity (ABTS at 85.2 μmol trolox/g and DPPH at 92.7 μmol trolox/g, respectively; *p* < 0.05) and 15% increase in total phenolic content (8.24 mg gallic acid/g) compared to non-fermented counterparts. The PHB formulation also enhanced color stability (lightness, redness, yellowness), textural characteristics (hardness, springiness, cohesiveness), and sensory acceptability. Volatile compound analysis revealed a reduction in off-odor aldehydes (hexanal, (E)-2-octenal, (E)-2-decenal, and (E,E)-2,4-decadienal) and increased production of desirable aromatic compounds like tetramethyl-pyrazine. These findings highlight the potential of combining lactic acid bacteria fermentation with burdock powder to develop functional duck sausages with improved nutritional and sensory properties.

## Simple Summary

Duck meat, while nutritious, often suffers from undesirable flavors that limit consumer acceptance. Traditional fermentation methods can improve flavor but lack consistency and health benefits. This study introduces a novel approach by combining burdock powder, a rich source of bioactive compounds, with selected lactic acid bacteria (*Lactiplantibacillus plantarum* and *Lactobacillus helveticus*) to enhance duck sausage quality. The results demonstrate significant improvements in antioxidant capacity, flavor profile, and texture, while reducing off-odors. This innovation not only addresses sensory limitations but also integrates health-promoting properties, offering a functional meat product with potential applications in gourmet and health-focused food markets. The findings highlight the synergy between natural ingredients and targeted fermentation, paving the way for future functional food development.

## 1. Introduction

Duck meat is the third most important type of poultry meat after chicken and turkey, in vigorous demand in many areas of the world, especially in Asia. People consume duck meat because of its high nutritional value, including its complete essential amino acid composition, superior fatty acid composition with a high level of polyunsaturated fatty acids, and balance between omega-6 fatty acids and omega-3 [1]. Duck meat has the advantages of low fat, low cholesterol, and high protein, which can effectively resist various inflammations and aging [2]. Therefore, more and more researchers have focused on developing different varieties of value-added products based on duck meat [3].

Duck meat has a unique smell that may negatively affect some consumer acceptance. The application of fermentation to remove off-odor is a common strategy. Traditional fermented duck sausages are made by mixing minced duck breast, pig back fat, and salt, stuffing the blend into natural casings, and allowing spontaneous fermentation at ambient temperature for 4–7 days using indigenous microbes. However, the quality of spontaneously fermented meat products is unstable and sometimes has safety issues. So, the inoculation of meat with starter cultures has become necessary. The microbial community mainly produces the volatile flavor compounds of fermented foods. For example, Li et al. [4] found that *Pediococcus* and *Lactococcus* contributed the most to the volatile flavor formation of tilapia sausage. Li et al. [5] also found that inoculating mixed starter cultures of *Lactobacillus* and *Staphylococcus* could improve flavor in fermented sausages during ripening. These lactic acid bacteria accelerated the volatile flavor formation and inhibited the spoilage microorganisms in the fermented tilapia sausage. The fermentation with lactic acid bacteria may also improve the flavor of duck sausages.

Burdock (*Arctium lappa* L.) is a biennial herbaceous plant in the Asteraceae family with medicinal and edible values. The roots, stems, leaves, and seeds of burdock all have special healthy functions. Burdock is a good source of bioactive compounds associated with cancer and cardiovascular disease prevention and anti-inflammatory effects [6]. Polysaccharides are important constituents in burdocks and have various bioactivities. Our previous study obtained pectic polysaccharides with cellular antioxidant activity [7] and another neutral polysaccharide with immune regulatory and anti-inflammatory effects, which was positively correlated with its antioxidant capacity [8].

Phenolic compounds, especially flavonoids, are also abundant in burdock roots and have suitable antioxidant activities [9]. It was found that *L. plantarum* had good DPPH (2,2-diphenyl-1-picrylhydrazyl radical) and hydroxide radical scavenging activities [10]. Wang et al. [11] compared the in vitro antioxidant activities of seven lactic acid bacteria and found that *L. plantarum* CCFM8661 and *L. casei* CCFMI 56 had more vigorous activities than others. The incorporation of lactic acid bacteria fermentation and burdock into duck sausages improves its flavor and health-promoting activities.

In this study, eight strains (Debaryomyces hansenii, Latilactobacillus sake, L. helveticus, L. casei, L. plantarum, Lactobacillus acidophilus, Pediococcus pentosaceus, and Lacticaseibacillus rhamnosus) were evaluated for their ability to ferment duck sausages supplemented with burdock powder. These starter cultures were specifically selected based on their documented efficacy in meat fermentation [4,5], GRAS (generally recognized as safe) status, or inclusion in China’s approved food microbial list, and preliminary screening results demonstrating superior acidification rates and off-odor reduction capabilities in duck meat matrices. The effect of these starters and burdock powder addition on the flavor, antioxidant capacity, bioactive compounds, volatile substances, and sensory properties of duck sausages were investigated.

## 2. Materials and Methods

### 2.1. Chemicals

Standards (gallic acid and rutin), sodium hydroxide, sodium acetate, sodium carbonate, ferric chloride hexahydrate, potassium phosphate monobasic, sodium nitrite, aluminum nitrate, and Folin–Ciocalteu reagent were obtained from J&K Chemical Ltd. (Beijing, China). ABTS (2,2′-azino-bis-(3-thylbenzo-thiazolne-6-sulfonic acid), DPPH (2,2-diphenyl-1-picrylhydrazyl radical), and TPTZ (2,4,6-tri (2-pyridyl)1,3,5-triazine) were purchased from Sigma Aldrich (Shanghai, China). Trolox (6-hydroxy-2.5.7.8-tetramethyl-chroman-2-carboxylic acid) was obtained from Across Organics (Morris Plains, NJ, USA). The media MRS (Man Rogosa and Sharpe), YPD (peptone dextrose yeast), and agar were purchased from Baoruyi Biotechnology Co., Ltd. (Beijing, China). Ethanol, acetic acid, phosphoric acid, and hydrochloric acid were obtained from Shanghai Macklin Reagent Co., Ltd. (Shanghai, China). The table salt came from Suguo Supermarket (Nanjing, China). All solvents and reagents were of analytical grade.

### 2.2. Materials

Duck breast, pig back fat, and natural casings were purchased from an online shopping platform. Specifically, 5 batches of duck breast and pig back fat were purchased, each in 10 kg frozen packages. Duck breast and pig back fat were used after cold thawing, and the casing was used after being soaked in clean water for 15 min. Burdock roots were obtained from Xuzhou Yamazaki Agricultural Products Technology R&D Co., Ltd. (Peixian, Xuzhou, China; latitude 34°43, longitude 116°56) in 2021. The fresh burdock was washed and dried at 60 °C in the oven. After grinding, coarse burdock powder was sifted through a sieve of 200 mesh and placed at 0 °C for later use. Strains *Lactiplantibacillus plantarum* (preservation number: No.23701, from Xinjiang traditional milk pimples), *L. casei* (No.15956, from Xinjiang traditional milk pimples), *L. helveticus* (No.24356, from traditional fermented kimchi), and *L. sake* (No.23702, from traditional pickled grass carps) were provided by China General Microbiological Culture Collection Center (Beijing, China). Yeast was also from traditional pickled grass carp. *Pediococcus pentosaceus* and *L. acidophilus* were purchased from the China Center Industrial Culture Collection (Beijing, China), and *L. rhamnosus* was obtained from Chr’s Hansen. The preservation numbers (e.g., 15,956) correspond to the strain numbers in our laboratory collection. Strains were propagated in MRS broth (for bacteria) and YPD plates (for yeast) at 30 °C for 24–48 h under aerobic conditions. Strains were identified using 16S rRNA sequencing for bacteria and ITS sequencing for yeast. Lactic acid bacteria were inoculated in De Man-Rogosa-Sharpe Medium (MRS) and yeasts in peptone dextrose yeast agar (YPD) in anaerobic jars (Jinheng Experimental Instrument Factory, Nanjing, China). They were cultured in MRS at 37 °C for 24 h and YPD broth at 32 °C for 3 days until the concentration reached about 10^10^ CFU/mL. The cultured bacteria solution was centrifuged at 10,000 r/min and 4 °C for 5 min. The collected bacteria were washed 3–4 times with sterile saline and re-suspended to a concentration of 10^9^ CFU/mL to obtain the seed liquid for later use [12].

### 2.3. Preparation of Fermented Burdock Sausage Samples

Naturally, fermented duck sausages (N) were prepared according to a traditional formula (only meat percentages added up to 100%, and percentages of other ingredients were meat-related), including duck breast meat (60%), pig back fat (40%), and table salt (2%). The procedure for the preparation of fermented sausages included the following: (1) rinsing the thawed raw meat in advance, mincing lean meat, and dicing the fat; (2) adding table salt to marinate for 2 h; (3) inoculating 2% seed liquid of different starters; (4) carrying out enema after chopping and mixing evenly; and (5) fermenting at constant temperature of 37 °C for one to four days and cooling to room temperature to obtain the product. The duck sausage was prepared by fermentation of the eight strains mentioned without adding burdock powder. The original naturally fermented duck sausage (N) without burdock powder and artificial inoculation was used as the blank control group for subsequent experiments. The group composed of sausages fermented with burdock powder (B) was obtained by adding 3% burdock powder. Seven starter groups were prepared, including *L. plantarum* (PB); *L. casei* (CB); *L. helveticus* (HB); *L. plantarum* and *L. casei* (1:1, PCB); *L. plantarum* and *L. helveticus* (1:1, PHB); *L. casei* and *L. helveticus* (1:1, CHB); and *L. plantarum*, *L. casei*, and *L. helveticus* (1:1:1, PCHB). Fermentation was carried out at 25 °C and 85% relative humidity (RH) for 48 h, followed by drying at 15 °C and 75% RH for 21 days. RH was controlled using a humidity chamber (Model: HCP-150, Memmert, Germany)**.** To ensure safety, spoilage and pathogenic microorganisms were enumerated using selective media, e.g., *Listeria monocytogenes* on PALCAM agar, *E. coli* on EMB agar, and *Salmonella* spp. on XLD agar.

### 2.4. Sensory Evaluation

Ten experienced food researchers of both sexes conducted a sensory evaluation test using a 10-point ranking scale (where 1 = least preferred and 10 = most preferred) as described by Gomezulu et al. [13]. The study was reviewed and approved by the Institution Review Board (IRB) of Jiangsu Academy of Agricultural Sciences (Nanjing, China), and informed consent was obtained from each panelist before participating in the study. The sausages fermented by eight strains and naturally fermented duck sausages at a constant temperature of 37 °C for 1–4 days were steamed in a steamer (Zhejiang Shaoxing Supor Home Appliance Manufacturing Co., Ltd., Hangzhou, China) or baked in an oven (Jiawei Road (China) Household Appliances Co., Ltd., Guangdong, China). The sausages were sliced into uniform sizes of about 2 cm in length and coded with 3-digit random numbers. Nine samples were served to panelists on white disposable plates in a randomized order. The panelists were asked to test and score according to their preference using the scale provided. Burdock duck sausages fermented for two days with a mixture of *Lactobacillus casei*, *L. helveticus*, and *L. plantarum* were grilled in an oven. The same measures were used for the other group.

### 2.5. Color Difference Analysis

Colorimeter (Keli Electronic Technology Co., LTD, Nanjing, China) was used to analyze the color value of nine groups of samples processed. The instrument was calibrated with a standard plate before measurement. For the measurement, each sample was cut into pieces with a thickness of about 1 cm. One piece was obtained for each treatment, and 5 points were measured for each piece. The lightness L* (0 = darkness, 100 = lightness), redness a* (+60 = red, −60 = green), and yellowness b* (+60 = yellow, −60 = blue) were measured and analyzed.

### 2.6. Texture Analysis

Texture profile analysis (TPA) was performed using a TA.XT Plus Texture Analyzer (Stable Micro Systems, Surrey, UK) equipped with a 5 kg load cell and a P/36R cylindrical probe. Each group of sausages was cut into about 2 cm slices. The sausage slice was placed in a texture tester and tested five times. Texture analyzer parameters were set as a flat-bottomed probe of P/100, pre-test rate at 1 mm/s, test rate at 1 mm/s, post-test rate at 1 mm/s, compression degree of 50%, and dwell time between two compressions of 5 s.

### 2.7. Extraction

A 2 g sample was weighed, and 20 mL of methanol aqueous solution (60%) was added for ultrasonic extraction with vortex mixing for 1 h, followed by centrifugation at 4000 r/min for 5 min. The fat was extracted with n-valeraldehyde and then filtered with 0.45 μm needle membrane for later use [14].

### 2.8. Detection of the Total Phenolics and Total Flavonoids

Total phenolic content (TPC) was carried out using the method previously described [9]. A final volume (4 mL) of the mixture of the sample solution, Folin–Ciocalteu reagent, 20% Na_2_CO_3_, and distilled water was incubated for 2 h. Absorbance was measured at 760 nm. The standard curve equation of gallic acid was obtained as *y* = 1.19*x* + 0.14 (*R*^2^ = 0.996). Results are expressed as micrograms of gallic acid equivalent per gram of dry weight (mg gallic acid/g).

Total flavonoid content (TFC) was measured according to reported methods [15]. The final volume (10 mL) of the mixture of the sample solution, 5% NaNO_2_, 10% Al(NO_3_)_3_, 4% NaOH, and distilled water were incubated for 10 min. Absorbance was measured at 509 nm. The standard curve equation of rutin was *y* = 2.63*x* + 0.04 (*R*^2^ = 0.999). Results are expressed as micrograms of rutin equivalent per gram of dry weight (mg rutin/g).

### 2.9. Antioxidant Capacity of Fermented Burdock Sausage Samples

Antioxidant capacity (DPPH and ABTS) was measured following the methodologies reported, with slight modifications [16]. The DPPH assay was performed by adding 150 µL of DPPH stock solution to 50 µL of each sample. After 30 min of incubation at 37 °C, absorbance was read at 517 nm using a 96-well ELX800 microplate reader (Tecan, Männedorf, Switzerland). For the ABTS assay, a stock solution (7 mM ABTS and 2.45 mM K_2_S_2_O_8_) was prepared and kept at room temperature for 12 h. The stock solution was then adjusted to an absorbance of 0.70 ± 0.02 at 734 nm. An amount of 50 µL of sample and 150 µL of ABTS were incubated for 6 min and read at 734 nm using the same microplate reader. Standard curves of ABTS free radical scavenging ability and DPPH radical scavenging ability were drawn using OD values and different Trolox solution concentrations as coordinate axes. The standard curve equation of ABTS and DPPH radical scavenging ability was *y* = −0.27*x* + 0.48 (*R*^2^ = 0.990) and *y* = −0.57*x* + 0.61 (*R*^2^ = 0.991).

Ferric ion-reducing antioxidant power (FRAP) was determined according to a method described [17]. A stock orange solution (20 mM of FeC1_3_^.^6H_2_O, 10 mM of TPTZ, and 0.30 mM of sodium acetate buffer) was prepared and kept in a constant-temperature water bath (Jiangsu Xinchunlan Scientific Instrument Co., Ltd., Changzhou, China) at 37 °C for 30 min. Briefly, 5 μL of the appropriately diluted sample was added to 180 μL of FRAP solution. Then the solution was incubated at 37 °C for 10 min, and the absorbance was read at 593 nm. The group without the sample solution was used as the control, and the group without the reaction solution was used as the blank. Different concentrations of Trolox solution (0, 0.2, 0.4, 0.6, 0.8, and 1.0 mM) were detected to draw the standard curve. The standard curve of FRAP was calculated and drawn using iron ion reduction capacity and different trolox solution concentrations as axes. The equation is *y* = 0.31*x* − 0.02 (*R*^2^ = 0.992). Results of the three antioxidant capacity assays were all expressed as micromole Trolox equivalent per gram of dry weight (μmol trolox/g).

### 2.10. Determination of Volatile Flavor Substances by HS-SPME-GC-MS

Volatile compounds in the natural fermentation group (N) and PHB group were analyzed by HS-SPME (headspace solid-phase microextraction) coupled with GC-MS (gas chromatography–mass spectrometry, Agilent 8890-5977B, Agilent Technologies, Inc., Santa Clara, CA, USA) [18]. An amount of 2 g of processed fermented sausage sample was placed in a sample vial (15 mL) and sealed with a sealing gasket. Then it was placed in a constant temperature water bath at 75 °C for 5 min. Volatiles were extracted by a SPME fiber (50/30 μm DVB/CAR/PDMS, Supelco, Inc., Bellefonte, PA, USA) in 75 °C water bath for 40 min and were desorbed in the gas chromatography injector for 5 min.

Volatile compounds were separated on a DB-WAX capillary column (30 m × 0.25 mm × 0.5 μm), and the chromatographic program was carried out. GC-MS data processing was automatically searched through the NIST11.L spectral library configured by the instrument. More than 80% matching data were selected, and the relative content of each volatile flavor component was determined by peak area normalization.

### 2.11. Statistics

Each experiment was performed at least in triplicate. Data are presented as mean value ± standard deviation (SD). The software GraphPad Prism 8.0.2 was used for figures and IBM SPSS Statistics 23 for data statistical analysis, with *p* < 0.05 representing a significant difference between means. Principal component analysis (PCA) was performed using the IBM SPSS Statistics 23 software and performed based on dimension reduction. The Pearson analysis used OriginPro 2022 software (OriginLab, Northampton, MA, USA). The Pearson correlation coefficient measured a linear relationship which was used for analyzing the correlation.

## 3. Results and Discussion

### 3.1. Selection of Candidate Strains for Combination Starter Cultures

Optimal starter cultures and fermentation time were selected by sensory evaluation (Table 1). People prefer baked fermented sausages compared with steamed fermented sausages. The sensory score was 26.84 for the baked sausage fermented by *P. pentosaceus* but only 16.34 for the steamed one, showing a significant difference between the baking and steaming processes. So, *P. pentosaceus* was not a suitable candidate strain for combination starter cultures. The total scores of sensory evaluation of duck sausage fermented by *L. casei*, *L. helveticus*, and *L. plantarum* were 26.62, 25.72, and 25.98, respectively, after steaming (*p* > 0.05), while their total scores after baking were 26.84, 26.36, and 26.06 (*p* > 0.05), respectively, which were approximate to the total sensory evaluation scores of sausages fermented by *L. rhamnosus*. This commercial probiotic strain was reported to be able to improve the quality of sausages used as positive control [19]. Therefore, *L. casei*, *L. helveticus*, and *L. plantarum* were selected as candidate strains to design the starters for the following duck sausage fermentation with nutritious burdock powder as an added ingredient. They are all on the list of strains that can be used in food at the China National Center for Food Safety Risk Assessment.

In this work, the fermentation time and temperature for the highest sensory evaluation score of fermented duck sausages was 2 days at 37 °C. This process was faster than the naturally fermented duck sausage, in which the fermentation time was 4 days. The result was consistent with the report on tilapia sausages that the addition of *P. pentosaceus* reduced the fermentation time from 48 h (naturally fermented tilapia sausage) to 24 h [4]. Wang et al. [5] and Li et al. [20] found that the inoculation of starter cultures resulted in a more substantial and quicker acidification of fermented sausages than that of spontaneous fermentation, which contributed to the improvement in the safety and flavor of meat products.

### 3.2. Sensory Evaluation for Selection of Optimal Starter Combination

Seven groups of sausages fermented by different starter combinations of *L. casei*, *L. helveticus*, and *L. plantarum* were prepared to compare their sensory quality with the spontaneous duck sausage (N) and the group with 3% burdock powder addition (B). Duck sausages, with 3% burdock powder addition, fermented with all these three strains (PCHB), and *L. helveticus* and *L. plantarum* fermented with two strains (PHB), had higher comprehensive sensory evaluation scores than others (Table 2). There was no significant difference between the PHB and PCHB groups in the sensory evaluation scores (*p* > 0.05). This indicated that using suitable composite strains and the addition of burdock powder could improve the taste and flavor of duck sausages.

### 3.3. Color and pH Evaluation for Selection of Optimal Starter Combination

Color is the first impression that food gives people. Good color affects consumers’ appetite and is the main factor in buying the product. The variance analysis showed no significant difference in the L*-value of the nine sausage groups (*p* > 0.05, Table 2). The L*-value of the PHB group was 11.28 ± 0.81, which was not significantly different from that of other groups. The a*-value reflected the redness of the sausages. The a*-value in the PB group (3.42 ± 0.44) was significantly higher than control (*p* < 0.05) but not statistically different from PHB (3.34 ± 0.45) or CB (3.22 ± 0.08) groups. The b*-value in PHB (5.96 ± 0.80) showed no significant difference versus PCHB (5.00 ± 1.12) or PB (4.79 ± 0.06) (*p* > 0.05). Studies have shown that the L*, a*, and b*-values in fresh pork sausage ranged from 40 to 50, 5 to 12, and 5 to 15, respectively [21]. The properties of the burdock powder and the raw duck meat determined the color behavior of the fermented sausages. In this experiment, the L*-values of fermented duck sausages were lower than those of pork sausage, which could be due to the dark yellow color of burdock powder and the red color of duck meat.

Based on pH analysis, the pH values of the N and PHB groups were 4.56 ± 0.02 and 4.75 ± 0.03, respectively. This result confirmed that LAB mainly produces an accumulation of organic acids during sausage fermentation. The lowering of pH is an essential requirement and ensures hygienic stability.

### 3.4. Texture Evaluation for Selection of Optimal Starter Combination

Textural properties of the duck sausages are shown in Table 2. Treatments containing burdock powder had higher textural properties than the control group (N, *p* < 0.05). The results indicated that adding burdock powder significantly affected the textural characteristics of the duck sausages. Laguna et al. [22] found an increase in hardness when inulin (a soluble fiber, fructo-oligosaccharide) was used in cookie manufacture. Burdock powder contains high contents of inulin [6], which might contribute to the texture improvement effect of sausages.

The texture properties of starter fermented groups were significantly higher than the control (*p* < 0.05). This might be due to the lactic acid bacteria in the starters resulting in lower pH values of fermented sausages. The lower pH value could cause the denaturation of proteins in the meat [23]. Denaturation and solidification of meat proteins reduced the moisture content of sausages so that the structure of sausages became more and more dense. The dense structure resulted in higher values of hardness, springiness, cohesiveness, and chewiness. The hardness, springiness, and cohesiveness of sausages in the PHB group were significantly higher than in other groups (*p* < 0.05). This indicated that the combined fermentation of *L. plantarum* and *L. helveticus* had the most pronouncedly positive effect on the texture of the sausages. Surasani et al. [24] reported that the approximate ranges of sausage hardness, springiness, cohesiveness, and chewiness were 4–3000 g, 10–800 g∙cm, 0–60, and 4–3000, respectively. However, in the present study, all the groups had much lower hardness, springiness, cohesiveness, and chewiness than the report. The reason might be that the sausage prepared in this experiment had not been air-dried. The water content is crucial to the texture because the protein mobility, cross-linking, and water absorption are all influenced by moisture [25]. Yuan et al. [26] found that the hardness gradually decreased as the sausage extradite was soaked in water. The hardness, springiness, and cohesiveness of sausages in the PHB group were significantly higher than in other groups (*p* < 0.05). This indicated that the combined fermentation of *L. plantarum* and *L. helveticus* had the most pronouncedly positive effect on the texture of the sausages.

### 3.5. Total Phenolic and Flavonoid Content Evaluation for Selection of Optimal Starter Combination

**Figure 1:**
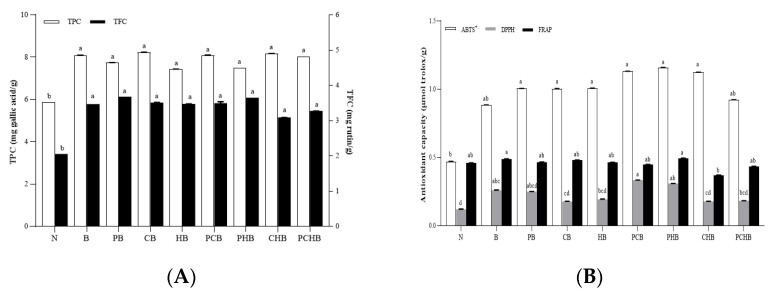
Comparison of total phenolic and flavonoid contents of burdock duck sausage fermented with different starter combinations. (**A**) Total phenolic content (TPC, white column) and total flavonoid content (TFC, black column); (**B**) ABTS free radical scavenging ability (white column); DPPH free radical scavenging ability (gray column); ferric ion reducing antioxidant power (black column). All the data were expressed as mean ± standard deviation (SD, n = 3). Different lowercase letters above the same color column indicate significant differences (*p* < 0.05). The group “N” means spontaneously fermented duck sausage without starter cultures and burdock powder. Groups designated as “B” had 3% burdock powder added; “PB” means B group was inoculated with *Lactiplantibacillus plantarum*; “CB” means B group was inoculated with *Lacticaseibacillus casei*; “HB” means B group was inoculated with *Lactobacillus helveticus*; “PCB” means B group was inoculated with *L. plantarum* and *L. casei* (1:1); “PHB” means B group was inoculated with *L. plantarum* and *L. helveticus* (1:1); “CHB” means B group was inoculated with *L. casei* and *L. helveticus* (1:1); “PCHB” means B group was inoculated with *L. plantarum*, *L. casei*, and *L. helveticus* (1:1:1).

As shown in Figure 1A, the total phenolic content (TPC, 7.44–8.24 mg gallic acid/g) and total flavonoid content (TFC, 3.09–3.68 mg rutin/g) in the groups containing burdock powder were all significantly higher than those in the spontaneous fermentation group (N, *p* < 0.05). This indicated that the addition of burdock powder and the starters could increase the content of these antioxidant substances. However, there was no significant difference among the groups of burdock duck sausages fermented with or without different starters (*p* > 0.05). The TPC values were 8.24, 7.49, and 7.44 mg of gallic acid/g in the CB, PHB, and HB groups, respectively. The highest total flavonoid content was found in the PB group (3.68 mg rutin/g), followed by the PHB and N groups (3.65 and 3.09 mg rutin/g, respectively). Filannino et al. also reported that lactic acid bacteria (*L. plantarum*, *Levilactobacillus spicheri, Limosilactobacillus fermentum,* and *Limosilactobacillus reuteri*) could bio-transfer the bioactive substances in plant cells into their metabolites and produce new phenolic compounds [27]. The low content of total phenolic in the PHB group might be due to the weaker ability of *L. plantarum* to bio-transform phenolic compounds [28]. During the process of bio-transformation, complex phenolic compounds might be hydrolyzed into simpler forms by hydrolytic enzymes of LAB strains [29]. The bioactive compounds in burdock could also be considered a key factor that affected the increase in TPC/TFC [6].

### 3.6. Antioxidant Capacity Evaluation for Selection of Optimal Starter Combination

ABTS and DPPH radical scavenging ability in the spontaneous fermentation group (N) was lower than those in burdock duck sausages fermented with or without different starters (Figure 1B), indicating that the addition of burdock powder could significantly increase the antioxidant activity of sausages. However, the ferric ion reducing antioxidant power (FRAP) demonstrated almost no significant difference, which might be because the sausages of all groups contained similar amounts of substances that could reduce Fe^3+^ to Fe^2+^. Generally, the PHB group had higher ABTS and DPPH radical scavenging ability and FRAP values than other starter-fermented groups. The higher antioxidant capacity of the PHB group compared with other different starter-combination-fermented groups was mainly due to the presence of *L. plantarum* and *L. helveticus*, which were confirmed to have strong antioxidant capacity in the previous report [30]. In addition, the burdock powder possessed antioxidant activity [9], which should also contribute to some antioxidant capacity in PHB here.

There was a significant correlation between the antioxidant activity and the content of polyphenols and flavonoids [25]. In this study, the change trend in antioxidant capacity was consistent with that in TPC or TFC, especially for the ABTS assay. The ABTS assay has been widely used to assess the total antioxidant capacities of crude extracts, which has been confirmed to exhibit a highly positive linear correlation with TPC [31]. Therefore, phenolics, especially flavonoids in sausages, were the primary contributors to the antioxidant activity.

### 3.7. Volatile Substance Difference Between Spontaneously Fermented (N) and Combination-Starter-Fermented (PHB) Burdock Duck Sausages

**Figure 2:**
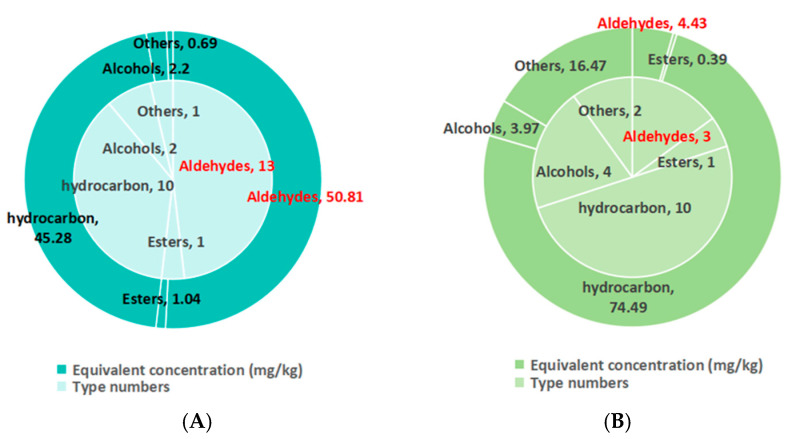
Comparison on type numbers and equivalent concentration of volatile substances in spontaneously fermented (**A**) and combination-starter-fermented (**B**) burdock duck sausages.

The PHB group is the best group of starter-combination-fermented sausages, and this process was able to improve the quality and flavor of sausages, including their sensory evaluation, color, texture, phenolic and flavonoid content, and antioxidant capacity. Therefore, the volatile substance of this group was further analyzed and compared with the spontaneously fermented duck sausages (N). A total of 27 volatile substances were identified in the N group, including 13 aldehydes (48.15%), 10 hydrocarbons (37%), 1 ester (3.7%), 2 alcohols (7.4%), and 1 other compound (3.7%). On the other hand, 20 volatile compounds were identified in the PHB group, including 3 aldehydes (15%), 10 hydrocarbons (50%), 1 ester (5%), 4 alcohols (20%), and 2 other compounds (10%) (Table 3). Compared to the N group, 14 odorants (including 11 aldehydes, 2 hydrocarbons, and 1 alcohol) disappeared, and 7 additional odorants (including 1 aldehyde, 2 hydrocarbons, 3 alcohols, and 1 other) appeared in the PHB group. The primary volatile substances in the N and PHB groups were aldehydes and hydrocarbons, respectively (Figure 2). Due to the low thresholds of aldehydes and high thresholds of hydrocarbons, aldehydes were identified as major odor-active compounds in foods. Since aldehydes contribute more significantly to perceived aroma (even at low concentrations) compared to hydrocarbons, their presence or absence directly influences flavor intensity. From Figure 2 and Table 3 and Table 4, we suspected that there was a strong flavor in the N group and a medium flavor in the PHB group. The result was consistent with the result of sensory evaluation. The PHB group had the highest sensory score, and the N group had a relatively lower sensory score.

Volatile compounds have different thresholds, so the relative content cannot reflect the true contribution that every volatile compound has made to the whole odor profile. The relative odor activity value (ROAV) has been proposed to evaluate the contribution of individual volatile compounds to the overall odor by its equivalent concentration [32,33]. The higher the ROAV, the higher its contribution to the whole aroma, as shown in Table 4. Seven aroma-active compounds with ROAV ≥ 1 were selected from the N. They were hexanal, (E)-2-octenal, (E)-2-nonenal, nonanal, (E,E)-2,4-decadienal, (E,E)-2,4-nonadienal, and (E)-2-decenal. Among them, hexanal was identified as a predominant odor-active compound with the highest ROAV value. It was usually reported as possessing the odor of grass and leaves [34] but was also considered to be one of the characteristic flavor compounds when duck aging occurred [35]. (E)-2-Nonenal, (E, E)-2,4-decadienal, and (E, E)-2,4-nonadienal delivered a fatty smell and have been identified as the primary odorants of chicken; they can be formed by the autoxidation of linoleic acid and arachidonic acid [36]. All of these seven aroma-active compounds belong to aldehydes, including alkyl aldehydes, enaldehydes, and dienaldehydes. They were products of the hydroperoxide degradation of linoleate and linolenic ester and usually have a great influence on the flavor of meat products due to their high content and low odor threshold [35,37,38,39].

Interestingly, hexanal, (E)-2-octenal, (E)-2-nonenal, (E,E)-2,4-decadienal, (E, E)-2,4-nonadienal, and (E)-2-decenal, which are closely related to duck off-flavor, had completely disappeared in PHB. It seemed that *L. plantarum* and *L. helveticus* could remove aldehydes by degrading off-odor compound precursors. These findings were in agreement with previous reports. Montanari et al. [40] found that *L. helveticus* has a possible pathway for linoleic conversion and oxylipin formation. During walnut fermentation, *Weissella cibaria* and *Leuconostoc mesenteroides* could release hydroxy and epoxy fatty acids from oleic, linoleic, and linolenic fatty acids [41].

On the other hand, nonanal, decanal, tetramethyl-pyrazine, styrene, and toluene were major aroma-active compounds (ROAV ≥ 1) in the PHB group. Nonanal, with the highest ROAV value, is derived from oleic acid degradation and gives duck meat a pleasant citrus and rose aroma [42]. Decanal has a strong aldehyde, sweet orange, and orange aroma when diluted, with an oily aroma [43]. Tetramethyl-pyrazine, which only existed in PHB, might have come from adding burdock powder, since methylpyrazines have been reported as contributors to burdock odor [33]. Toluene is widely used in flavor synthesis [44]. Due to the high threshold of hydrocarbon, the impact of styrene and toluene on the overall flavor of food is minimal. However, styrene and toluene, which were also found in sauced ducks, still contributed to the aroma characteristics of *L. plantarum*, *L. helveticus,* and burdock powder fermented duck sausage.

## 4. Conclusions

Incorporating burdock powder and lactic acid bacteria into duck sausages could represent a good alternative for providing some added value to traditional meat products. The product PHB had better texture, redness and yellowness, antioxidant capacity, and sensory evaluation than other experimental products. Burdock powder and mixed strains (recombined *L. plantarum* and *L. helveticus*) could not only remove the undesirable flavor substances but also increase the flavor substances. As regards burdock powder, it could give sausages a special flavor and balance the nutrition of sausages. Burdock powder was also a source of dietary fiber and bioactive substances, e.g., polysaccharides, phenolics, and flavonoids. Future studies should investigate the impacts of burdock–starter combinations on shelf-life stability, particularly regarding microbial safety, lipid oxidation, and probiotic viability during storage, to assess commercial applicability.

## Figures and Tables

**Table 1:** Sensory evaluation scores of steamed or baked fermented burdock duck sausages with eight different strains.

	Time (Day)	1	2	3	4	SUM
Strains						
Steamed burdock duck sausages	Control	6.12 ± 0.66 ^aB^	7.10 ± 0.66 ^aA^	6.98 ± 0.26 ^aA^	7.14 ± 0.55 ^aA^	27.34 ± 1.40 ^a^
	*Lacticaseibacillus rhamnosus*	6.14 ± 0.37 ^aB^	7.20 ± 0.40 ^abA^	7.06 ± 0.20 ^aA^	6.76 ± 0.48 ^aA^	27.16 ± 0.80 ^a^
	*Lactobacillus* *helveticus*	5.66 ± 0.55 ^aB^	7.00 ± 0.45 ^abA^	6.44 ± 0.21 ^aA^	6.62 ± 0.47 ^aA^	25.72 ± 0.64 ^a^
	*Lacticaseibacillus casei*	6.34 ± 0.76 ^aA^	6.70 ± 0.75 ^bcA^	6.88 ± 0.21 ^aA^	6.70 ± 0.43 ^abA^	26.62 ± 1.77 ^a^
	*Latilactobacillus sake*	6.24 ± 0.41 ^aA^	6.24 ± 0.39 ^bcA^	6.44 ± 0.33 ^aA^	6.54 ± 0.23 ^aA^	25.46 ± 0.64 ^a^
	*Lactiplantibacillus plantarum*	6.32 ± 0.59 ^aA^	6.70 ± 0.75 ^bcA^	6.72 ± 0.42 ^aA^	6.24 ± 0.22 ^abA^	25.98 ± 0.80 ^a^
	*Lactobacillus* *acidophilus*	6.10 ± 0.42 ^aAB^	6.02 ± 0.63 ^bcB^	6.80 ± 0.40 ^aA^	6.60 ± 0.26 ^abAB^	25.52 ± 0.90 ^a^
	*Pediococcus pentosaceus*	5.70 ± 0.60 ^aA^	6.44 ± 0.39 ^cA^	2.20 ± 0.75 ^bB^	2.00 ± 0.89 ^bcB^	16.34± 1.66 ^b^
	*Debaryomyces hansenii*	6.30 ± 1.33 ^aA^	6.60 ± 0.58 ^bcA^	5.76 ± 0.69 ^bA^	6.88 ± 0.19 ^cA^	25.54 ± 1.31 ^a^
Baked burdock duck sausages	Control	7.72 ± 0.70 ^aA^	6.20 ± 0.24 ^cdfB^	7.62 ± 0.35 ^aA^	6.72 ± 0.38 ^aB^	28.26 ± 0.73 ^ab^
	*Lacticaseibacillus rhamnosus*	7.54 ± 0.70 ^aAB^	7.80 ± 0.81 ^aA^	7.42 ± 0.43 ^abAB^	6.64 ± 0.55 ^aB^	29.40 ± 1.66 ^a^
	*Lactobacillus* *helveticus*	7.20 ± 1.03 ^abA^	6.60 ± 0.58 ^bcdA^	6.48 ± 0.55 ^abcA^	6.08 ± 0.55 ^aA^	26.36 ± 1.29 ^bc^
	*Lacticaseibacillus casei*	6.64 ± 0.42 ^abAB^	7.46 ± 0.82 ^abA^	5.98 ± 0.84 ^cB^	6.76 ± 0.64 ^aAB^	26.84 ± 1.30 ^bc^
	*Latilactobacillus sake*	6.80 ± 0.51 ^abA^	5.50 ± 0.89 ^fAB^	4.70 ± 1.99 ^dB^	6.48 ± 0.93 ^aAB^	23.48 ± 2.67 ^d^
	*Lactiplantibacillus plantarum*	7.16 ± 0.90 ^abA^	6.20 ± 0.81 ^cdfA^	6.24 ± 0.67 ^bcA^	6.46 ± 0.83 ^aA^	26.06 ± 1.28 ^bc^
	*Lactobacillus* *acidophilus*	6.52 ± 0.79 ^abA^	5.90 ± 0.92 ^dfA^	6.10 ± 0.60 ^bcA^	6.74 ± 0.44 ^aA^	25.26 ± 1.06 ^cd^
	*Pediococcus pentosaceus*	6.62 ± 1.04 ^abA^	7.20 ± 0.40 ^abcA^	6.34 ± 0.31 ^abcA^	6.68 ± 0.72 ^aA^	26.84 ± 0.52 ^bc^
	*Debaryomyces hansenii*	6.02 ± 0.82 ^bB^	7.16 ± 0.21 ^abcA^	6.48 ± 0.45 ^abcAB^	5.92 ± 0.48 ^aB^	25.58 ± 1.81 ^cd^

The data are expressed as mean ± standard deviation (SD, n = 10). Different lowercase letters indicate significant differences within the same column in steamed or baked burdock duck sausages (*p* < 0.05). Different uppercase letters indicate significant differences within the same row in steamed and baked burdock duck sausages (*p* < 0.05).

**Table 2:** Sensory evaluation scores, color analysis, and texture analysis of burdock duck sausage fermented with different starters.

Group	Sensory Evaluation Scores	Color Analysis			Texture Analysis			
		Lightness	Redness	Yellowness	Hardness	Springiness	Cohesiveness	Chewiness
N	6.92 ± 0.78 ^ab^	9.25 ± 0.98 a	0.39 ± 0.23 b	1.79 ± 0.36 c	0.22 ± 0.15 c	0.05 ± 0.01 f	0.08 ± 0.04 c	0.02 ± 0.01 d
B	6.99 ± 0.45 ^ab^	12.59 ± 1.44 a	0.65 ± 0.41 b	2.35 ± 0.28 bc	0.40 ± 0.27 bc	0.70 ± 0.32 cde	0.22 ± 0.14 abc	0.20 ± 0.17 abcd
PB	6.67 ± 0.73 ^b^	10.03 ± 1.41 a	3.42 ± 0.44 a	4.79 ± 0.06 ab	1.09 ± 0.31 abc	1.05 ± 0.06 bc	0.48 ± 0.15 abc	0.49 ± 0.13 abc
CB	6.93 ± 1.03 ^ab^	9.31 ± 1.78 a	3.22 ± 0.08 a	4.48 ± 0.55 abc	0.20 ± 0.01 c	0.29 ± 0.04 ef	0.09 ± 0.01 c	0.03 ± 0.01 d
HB	6.83 ± 0.80 ^ab^	9.44 ± 2.37 a	2.95 ± 0.74 a	3.81 ± 1.39 abc	0.50 ± 0.03 bc	0.64 ± 0.01 cde	0.26 ± 0.02 abc	0.19 ± 0.01 bcd
PCB	7.06 ± 0.54 ^ab^	12.71 ± 3.00 a	2.25 ± 0.10 a	4.72 ± 0.65 ab	0.84 ± 0.62 abc	0.81 ± 0.20 bcd	0.36 ± 0.25 abc	0.35 ± 0.28 abcd
PHB	7.19 ± 0.60 ^a^	11.28 ± 0.81 a	3.34 ± 0.45 a	5.96 ± 0.80 a	1.72 ± 0.86 a	1.78 ± 0.24 a	0.59 ± 0.28 a	0.59 ± 0.14 ab
CHB	7.02 ± 0.66 ^ab^	9.45 ± 2.76 a	2.53 ± 0.04 a	4.10 ± 0.99 abc	1.21 ± 0.24 ab	1.21 ± 0.07 b	0.51 ± 0.10 ab	0.62 ± 0.15 a
PCHB	7.16 ± 0.49 ^a^	9.52 ± 3.62 a	2.43 ± 0.16 a	5.00 ± 1.12 ab	0.17 ± 0.02 c	0.43 ± 0.01 def	0.11 ± 0.01 bc	0.05 ± 0.003 cd

The data was expressed as mean ± standard deviation (SD, n = 10 for sensory evaluation, n = 5 for color analysis, n = 3 for texture analysis, respectively). Different lowercase letters indicate significant differences within the same column (*p* < 0.05). The group “N” means spontaneously fermented duck sausage without starter cultures and burdock powder. For the groups designated as “B”, 3% burdock powder was added; “PB” means B group was inoculated with *Lactiplantibacillus plantarum*; “CB” means B group was inoculated with *Lacticaseibacillus casei*; “HB” means B group was inoculated with *Lactobacillus helveticus*; “PCB” means B group was inoculated with *L. plantarum* and *L. casei* (1:1); “PHB” means B group was inoculated with *L. plantarum* and *L. helveticus* (1:1); “CHB” means B group was inoculated with *L. casei* and *L. helveticus* (1:1); “PCHB” means B group was inoculated with *L. plantarum*, *L. casei*, and *L. helveticus* (1:1:1).

**Table 3:** Comparison of major volatile substances between original duck sausages and burdock duck sausages fermented with *Lactiplantibacillus plantarum* and *Lactobacillus helveticus*.

Compound	CAS	LRI	Aroma Threshold (μg/L)	Equivalent Concentration (mg/kg)		ROAV	
				N	PHB	N	PHB
Aldehydes							
Hexanal	66-25-1	802	0.32	13.95	-	100	-
2-Heptenal, (E)-	18829-55-5	960	34	3.20	-	0.22	-
Benzaldehyde	100-52-7	971	24	4.44	-	0.42	-
Octanal	124-13-0	1006	7	2.75	-	0.9	-
Benzeneacetaldehyde	122-78-1	1053	4	6.67	-	3.82	-
Nonanal	124-19-6	1102	2.8	11.52	3.60	9.43	100
2-Nonenal, (E)-	18829-56-6	1166	0.15	1.24	-	18.98	-
2-Octenal, (E)-	2548-87-0	1063	3	2.17	-	1.66	-
2,4-Nonadienal, (E,E)-	5910-87-2	1210	0.1	0.24	-	5.47	-
2-Decenal, (E)-	3913-81-3	1262	0.3	0.92	-	7.00	-
Decanal	112-31-2	1175	1.97	0.81	0.39	0.95	15.49
2,4-Decadienal, (E,E)-	25152-84-5	1317	0.2	0.81	-	9.34	-
14-Octadecenal	56554-89-3	1863	-	2.09	-	-	-
Hexadecanal	629-80-1	1830	-	-	0.44	-	-
Esters							
Octadecanoic acid, phenylmethyl ester	5531-65-7	2794	-	1.04	0.39	-	-
Hydrocarbon							
Toluene	108-88-3	774	1000	11.31	20.77	0.00	1.62
p-Xylene	106-42-3	865	530	2.62	3.77	0.01	0.55
Styrene	100-42-5	893	50	16.47	18.19	0.76	1.94
Decane	124-18-5	1000		-	9.74	-	0.08
Undecane	1120-21-4	1100	10,000	3.07	17.05	0.00	0.13
Undecane, 3-methyl-	1002-43-3	1170	-	0.51	1.08	-	-
Tetradecane	629-59-4	1400	1000	2.18	2.43	0.00	0.19
Tetradecane, 2,6,10-trimethyl-	14905-56-7	1539	-	-	0.30	-	-
Nonadecane	629-92-5	1900	-	0.71	0.79	-	-
Octane, 2,7-dimethyl-	1072-16-8	928	-	7.14	-	-	-
Cyclohexene, 3-(2-methylpropyl)-	4104-56-7	1001	-	0.72	-	-	-
Octadecane, 6-methyl-	10544-96-4	1842	-	0.55	0.37	-	-
Alcohols							
1,2-Propanediol, 3-methoxy-	623-39-2	977	-	-	3.47	-	-
2-Propanol, 1-chloro-3-methoxy-	4151-97-7	904	-	1.51	-	-	-
2-Hexadecanol	14852-31-4	1702	-	-	0.05	-	-
1-Hexadecanol, 2-methyl-	2490-48-4	1870	-	-	0.19	-	-
Cedrol	77-53-2	1598	-	0.69	0.26	-	-
Others							
Pyrazine, tetramethyl-	1124-11-4	1089	1000	-	15.99	-	1.24
Formamide, N,N-dibutyl-	761-65-9	1310	-	0.69	0.48	-	-

“-“means the relative content was not detectable; “ROAV” means relative odor activity value; ”N” means spontaneously fermented duck sausages without starter cultures and burdock powder; ”PHB” means duck sausage fermented with *Lactobacillus helveticus* and *Lactiplantibacillus plantarum* with the addition of burdock powder.

**Table 4:** Comparison of main odor-active compounds (ROAV ≥ 1) in the spontaneously fermented duck sausages and burdock duck sausages fermented by *Lactobacillus helveticus* and *Lactiplantibacillus plantarum*.

Compound	Odor Descriptor	ROAV		
		N	PHB	Change
Aldehydes				
Hexanal	Fresh green, leafy fruity, sweaty	100	-	↓
2-Octenal, (E)-	Fruity, aldehyde-like, fatty,	1.66	-	↓
Nonanal	Waxy, fresh orris, orange peel	9.43	100	↑
2-Nonenal, (E)-	Fatty, green	18.98	-	↓
2,4-Decadienal, (E,E)-	Orange, coriander, geranium, fatty	9.34	-	↓
Decanal	Soap, orange peel, tallow	<1	15.49	↑
2,4-Nonadienal, (E,E)-	Fatty, grassy	5.47	-	↓
2-Decenal, (E)-	Tallow, orange	7.00	-	↓
Hydrocarbon				
Styrene	Balsamic, gasoline	<1	1.94	↑
Toluene	Paint	<1	1.62	↑
Heterocyclic compounds				
Pyrazine, tetramethyl-	Roast, earth	-	1.24	↑

“ROAV” means relative odor activity value; “N” means spontaneously fermented duck sausages without starter cultures and burdock powder; “PHB” means duck sausage fermented by *L. plantarum* and *L. helveticus* with the addition of burdock powder. ↑: PHB group > N group in ROAV, ↓: PHB group < N group in ROAV.

## Data Availability

The data presented in this study are available upon request.
